# Supplementary material for: Renewed proliferation in adult mouse cochlea and regeneration of hair cells
Source: Nat Commun. 2019 Dec 4;10:5530. doi: 10.1038/s41467-019-13157-7 (PMC6892913; doi:10.1038/s41467-019-13157-7)
Supplement: Supplementary file 1 — Supplementary Information [file 41467_2019_13157_MOESM1_ESM.pdf]

SUPPLEMENTARY INFORMATION

Renewed proliferation in adult mouse cochlea and regeneration of hair cells

Shu et al.

SUPPLEMENTARY FIGURES

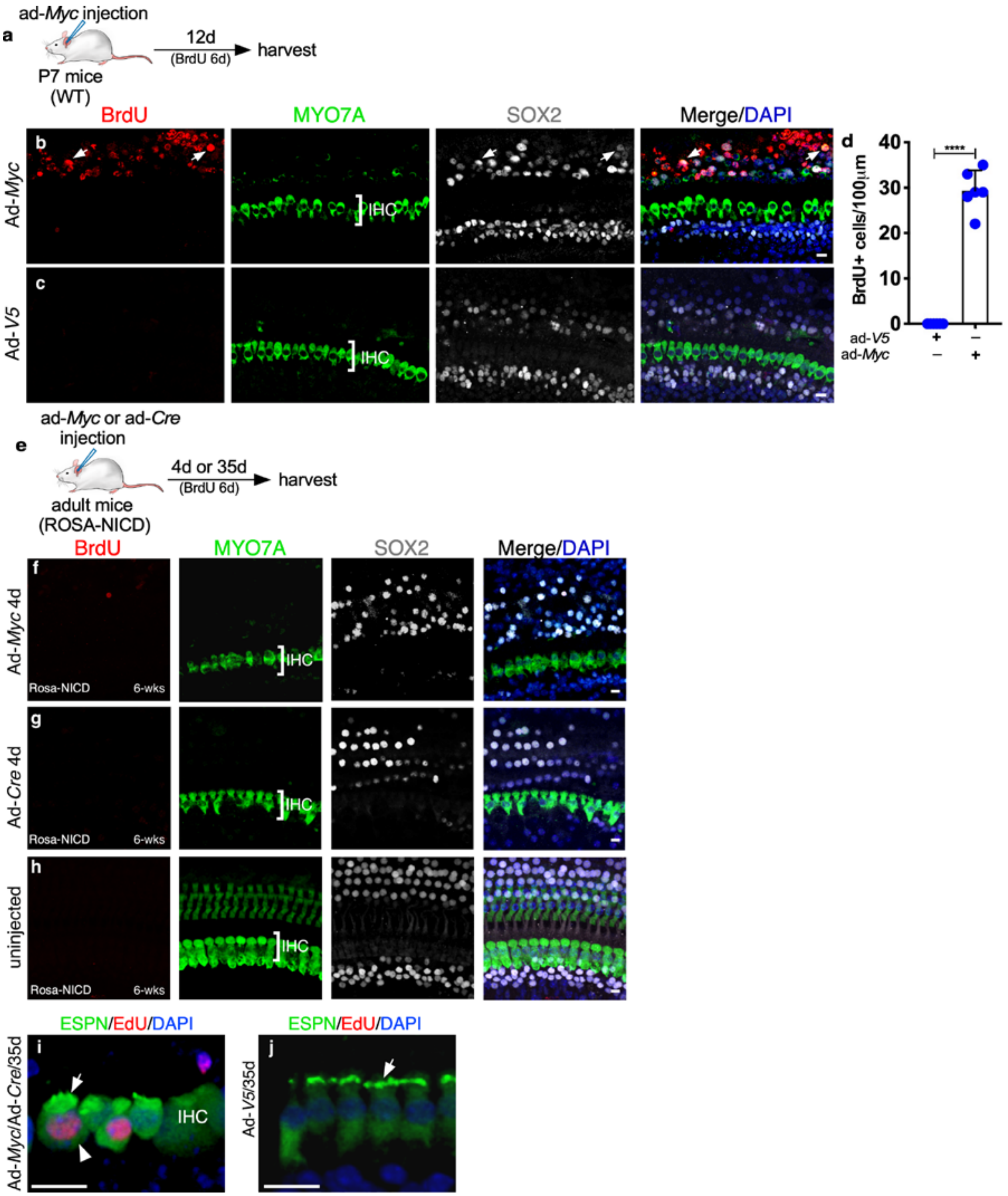

Supplementary Figure 1. Effect of *Myc/NICD* co-activation on proliferation of SCs and IHCs *in vivo*. (**a**) A diagram illustrating the experimental procedure of ad-*Myc* injection by cochleostomy into postnatal (P7) wild type (WT) cochlea. **(b)** Numerous SCs ($SOX2^{+}$) were labeled with BrdU (arrows) twelve days after injection. No HC was BrdU

positive. **(c)** Twelve days after control ad-*V5* injection into P7 WT mouse cochlea, no proliferating cells were detected. (**d**) Quantification and comparison of $BrdU^+$ SCs at the mid-base turn in P7 WT cochlea injected with ad-*Myc* and ad-*V5*. ****$p<0.0001$, two-tailed unpaired Student's t-test. Error bar, mean ± s.d.; n=6 biologically independent samples for each group. (**e**) A diagram illustrating the experimental procedure of ad-*Myc* or ad-*Cre* injection into adult Rosa-NICD cochlea by cochleostomy *in vivo*. **(f,g)** Four days after ad-*Myc* (**f**) or ad-*Cre* (**g**) *in vivo* injection into the adult Rosa-NICD cochleae, no proliferating cells were detected. (**h**) An uninjected cochlea of adult Rosa-NICD showed no proliferation. (**i**) 3D reconstruction of z-stacked confocal images of dividing IHCs ($ESPN^+/EdU^+$, arrowhead) with stereocilia structure (arrow) 35 days post ad-*Myc*/ad-*Cre* into adult (6-week-old) Rosa-NICD cochlea *in vivo*. (**j**) 3D reconstruction of z-stacked confocal images of control IHC stereocilia (arrow) 35 days after ad-*V5* injection into adult (6-week-old) Rosa-NICD cochlea *in vivo*. Scale bars: 10 μm.

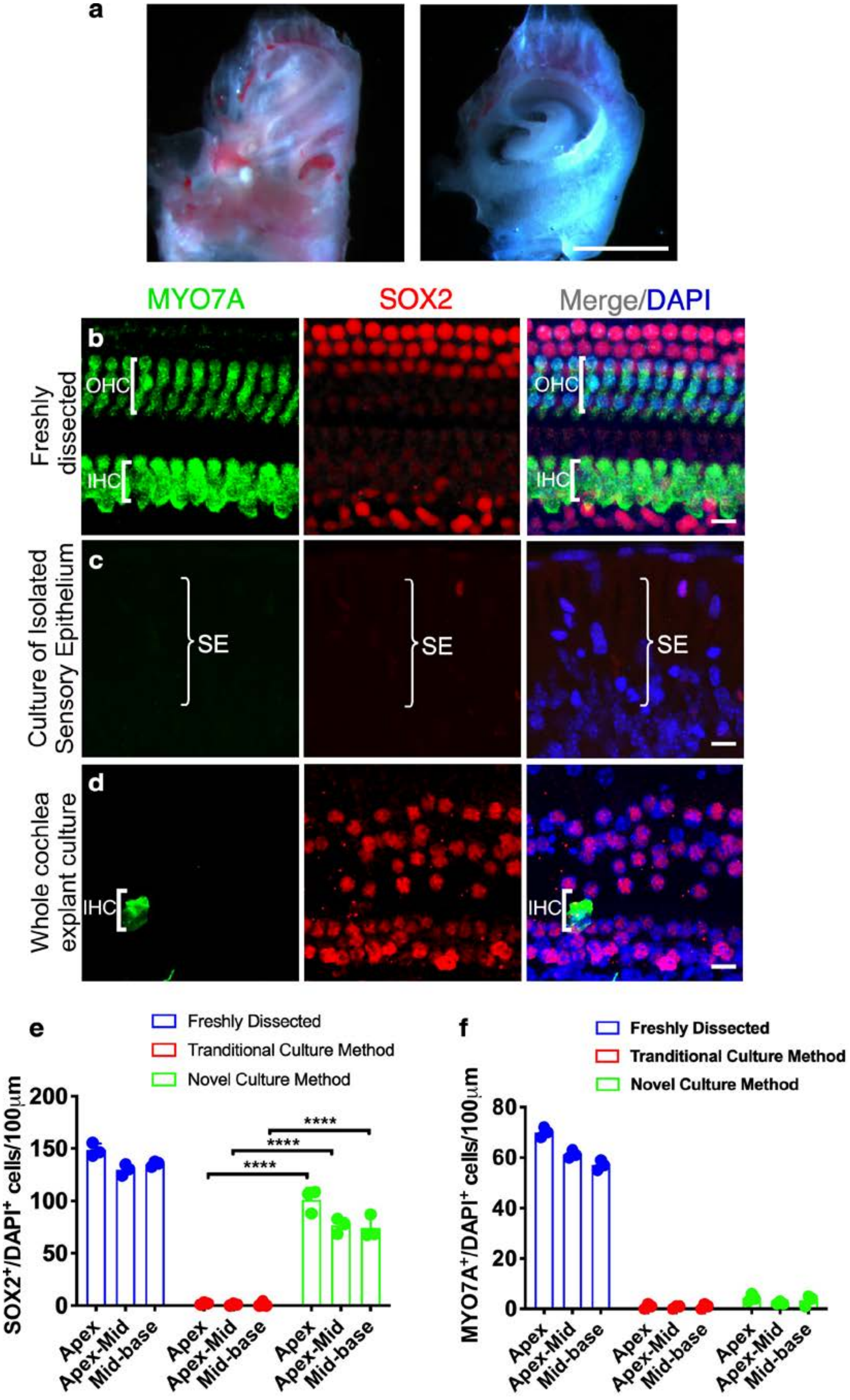
a
MYO7A
SOX2
Merge/DAPI
b
Freshly dissected
OHC
IHC
c
Culture of Isolated Sensory Epithelium
SE
d
Whole cochlea explant culture
IHC
e
Freshly Dissected
Tranditional Culture Method
Novel Culture Method
SOX2+/DAPI+ cells/100μm
200
150
100
50
0
****
Apex
Apex-Mid
Mid-base
f
MYO7A+/DAPI+ cells/100μm
80
60
40
20
0

Supplementary Figure 2. Establishment of adult cochlea explant culture. (**a**) A freshly dissected adult (6-week-old) wild type mouse cochlea with the intact bone (left) and with the bone from the apex removed (right) before culture. (**b**) Distribution of HCs and SCs in a freshly dissected cochlea of an adult mouse (6-week-old). (**c**) Culture of the adult cochlear sensory epithelium (SE) alone resulted in a complete loss of HCs and SCs one week later. (**d**) Adult whole cochlea explant culture with the bone maintained the overall structure and preserved most SCs ($SOX2^+$) with the loss of HCs (MYO7A) after two weeks. The mid-apical turn of cochlea was shown. (**e,f**) Quantification and comparison of SCs ($SOX2^+/DAPI^+$) cells (**e**) and HCs ($MYO7A^+/DAPI^+$ cells) (**f**) from adult cochleae under different conditions showed an overall preservation of SCs and the loss of HCs by the novel explant culture method. ****$p<0.0001$, two-way ANOVA with Tukey's multiple comparison test. Error bar, mean ± s.d.; n=3 biologically independent samples for each condition. Scale bars: 1 mm in a, 15 μm in others.

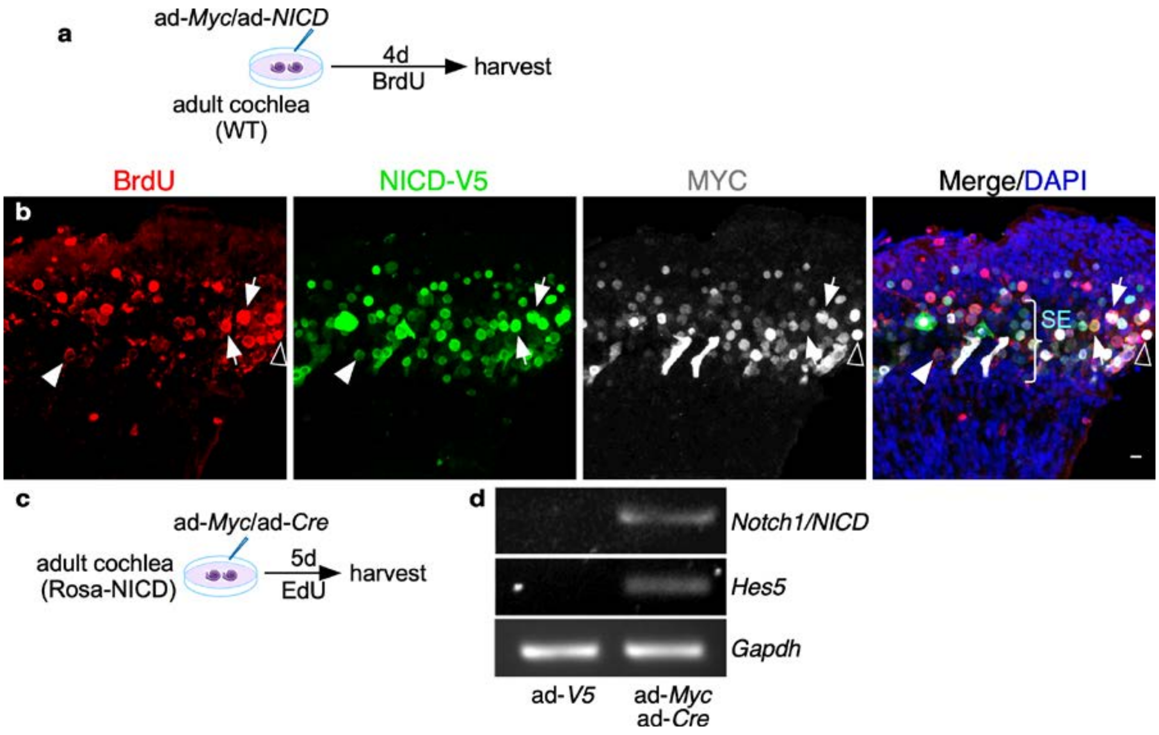

Supplementary Figure 3. **Proliferation in adult cochleae is the direct result of *Myc* and *NICD* co-activation**. (**a**) A diagram illustrating the procedure. (**b**) Five days post ad-*Myc*/ad-*NICD*-V5 infection in wild type adult (6-week-old) cochlea *in vitro*, the majority of the proliferating cells ($BrdU^+$) in the sensory epithelial region (SE, bracket) were positive for both MYC (anti-MYC antibody labeling) and NICD (detected by an anti-V5 antibody), shown by the arrows. Arrowhead, $BrdU^+$/ MYC^-/$NICD^+$ cell; open arrowhead, $BrdU^+$/MYC^+/$NICD^-$ cell. The area shown was from the mid-apical turn. (**c,d)** Semi-quantitative RT-PCR showed up-regulation of *NICD* and *Notch1* target *Hes5*, five days after ad-*Myc*/ad-*Cre* infection of the cultured adult (6-week-old) Rosa-NICD cochlea. Control was the ad-*V5*-infected cultured adult Rosa-NICD cochlea. Scale bars: 10 μm.

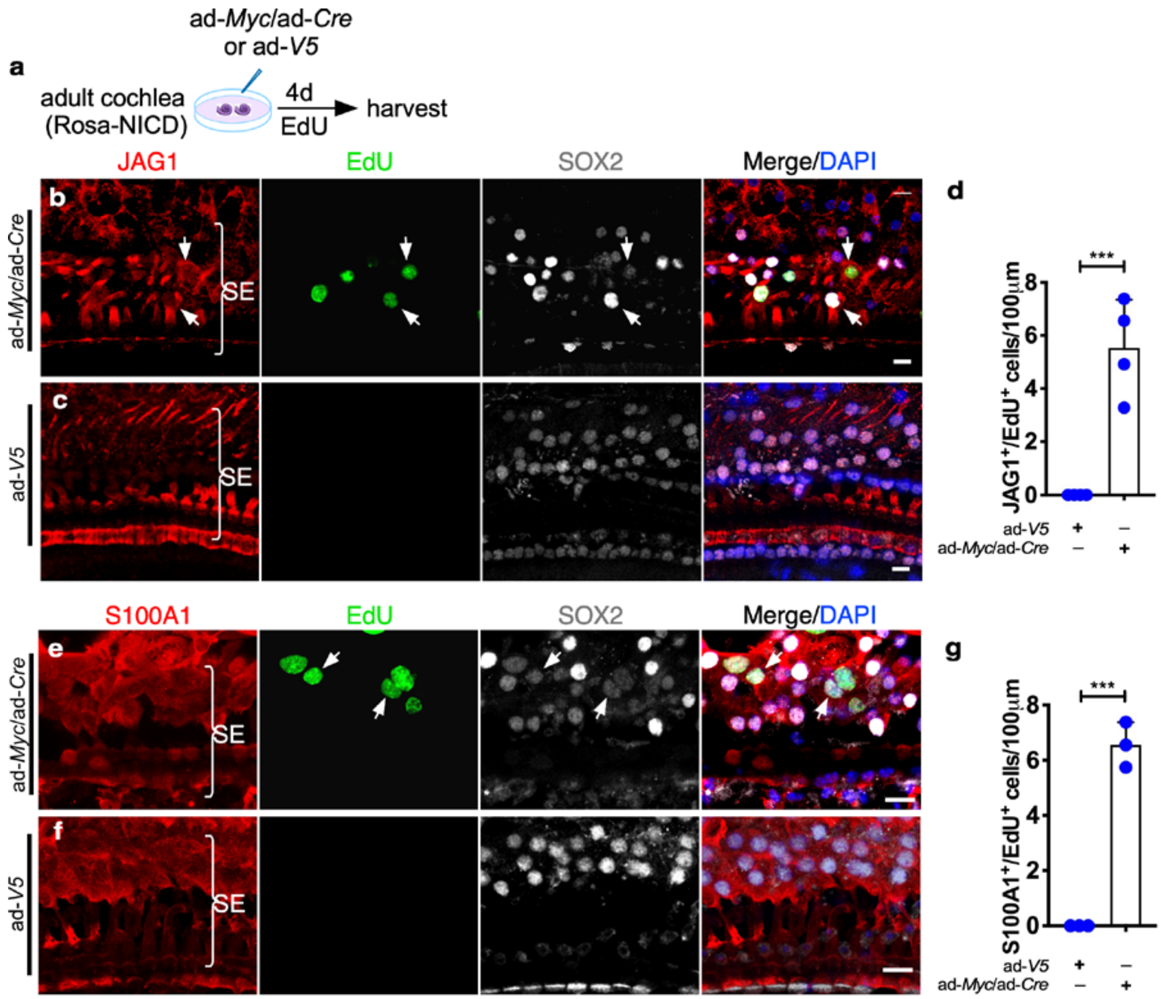

Supplementary Figure 4. Adult cochlear SC subtypes undergoing proliferation. (a) A diagram illustrating the procedure. (**b,c**) Dividing JAG1$^+$ SCs (JAG1$^+$/EdU$^+$, arrows) were seen four days after ad-*Myc*/ad-*Cre* infection of the cultured adult Rosa-NICD mouse cochlea (b). No EdU$^+$ cell was observed in control ad-*V5*-infected Rosa-NICD mouse cochlea (c). (**d**) Quantification showed a significant increase in JAG1$^+$/EdU$^+$ cells in the ad-*Myc*/ad-*Cre*-infected Rosa-NICD cochleae *in vitro*. ****p<0.001*, two-tailed unpaired Student's t-test. Error bar, mean ± s.d.; n=4 for each group. (**e,f**) Proliferating S100A1$^+$ SCs (S100A1$^+$/EdU$^+$, arrows) were detected four days after ad-*Myc*/ad-*Cre* infection of the cultured adult Rosa-NICD cochlea (e). No

proliferation was detected in ad-*V5* infected adult Rosa-NICD cochlea control (f). (**g**) Quantification of $S100A1^{+}/EdU^{+}$ cells in the ad-*Myc*/ad-*Cre*- and ad-*V5*-infected adult Rosa-NICD cochleae in culture. ***$p<0.001$, two-tailed unpaired Student's t-test. Error bar, mean ± s.d.; n=3 for each group. n is the number of biologically independent samples. Scale bars: 10 μm.

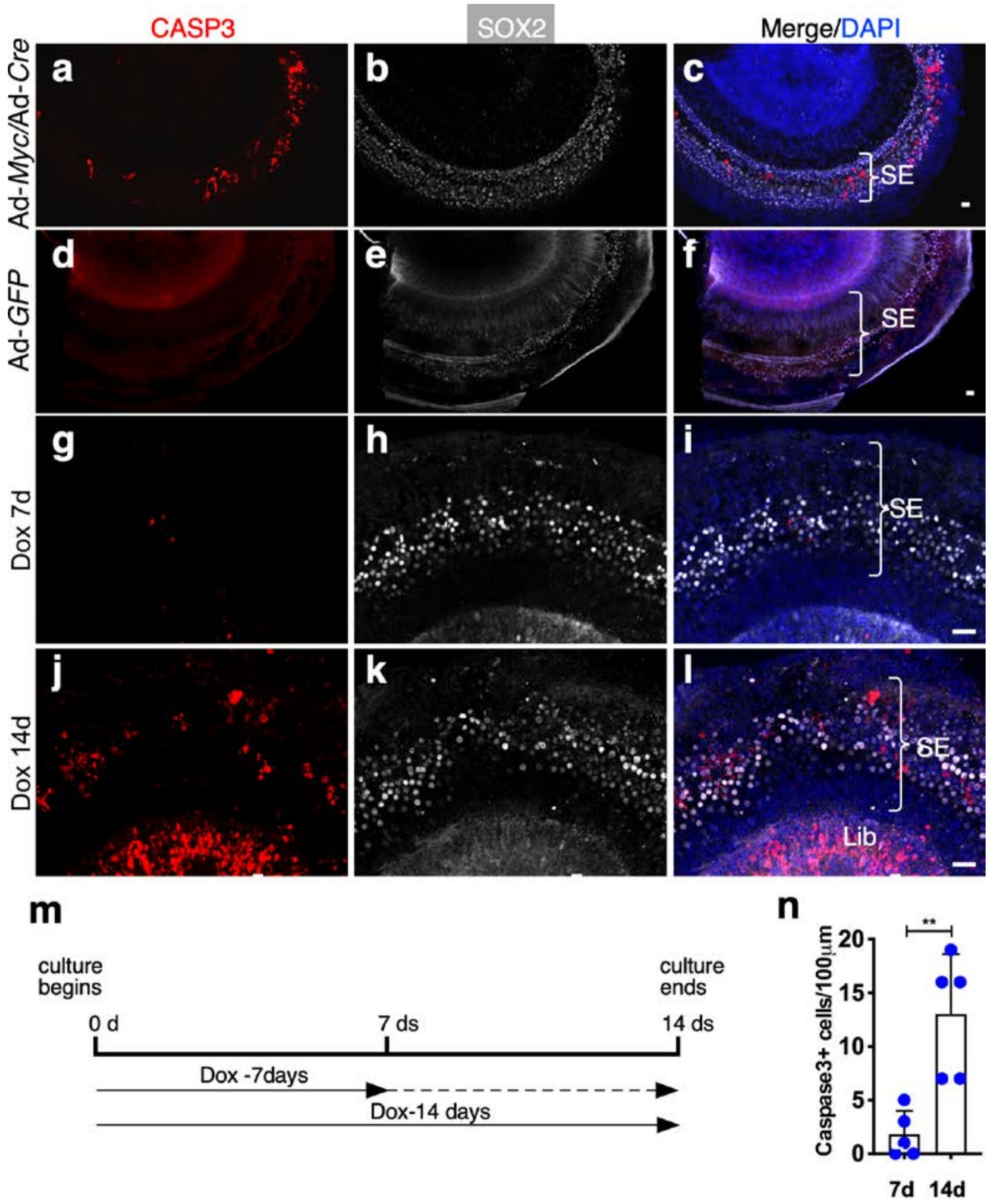

Supplementary Figure 5. ***Myc/NICD* activation and cell survival.** (**a-c**) Four days after ad-*Myc*/ad-*Cre in vitro* infection in cultured adult Rosa-NICD cochlea, increased CASP3$^+$ cells were seen in the sensory epithelial (SE) region of the mid-apical turn. (**d-f**) In the ad-*GFP*-infected adult Rosa-NICD cochlea control, virtually no CASP3$^+$ cell was detected in the SE region of the mid-apical turn. (**g-i**) Exposure to Dox for 7 days, adult (6-week-old) rtTA/tet-*Myc*/tet-*NICD* mouse cochlea showed few CASP3$^+$ cells in the SE

(bracket) in the mid-apical turn. (**j-l**) Exposure to Dox for 14 days, adult rtTA/tet-*Myc*/tet-*NICD* cochlea showed a dramatic increase in $CASP3^+$ cells in the SE (bracket) and the limbus region (Lib) in the mid-apex. (**m**) A schematic diagram of the experimental procedure in g-l. (**n**) Quantification showed a significant increase in $CASP3^+$ cells after 14-day compared to 7-day Dox exposure in cultured rtTA/tet-*Myc*/tet-*NICD* mouse cochleae. $**p<0.01$, two-tailed unpaired Student's t-test. Error bar, mean±s.d.; n=5 biologically independent samples for each group. Scale bars: 20 μm.

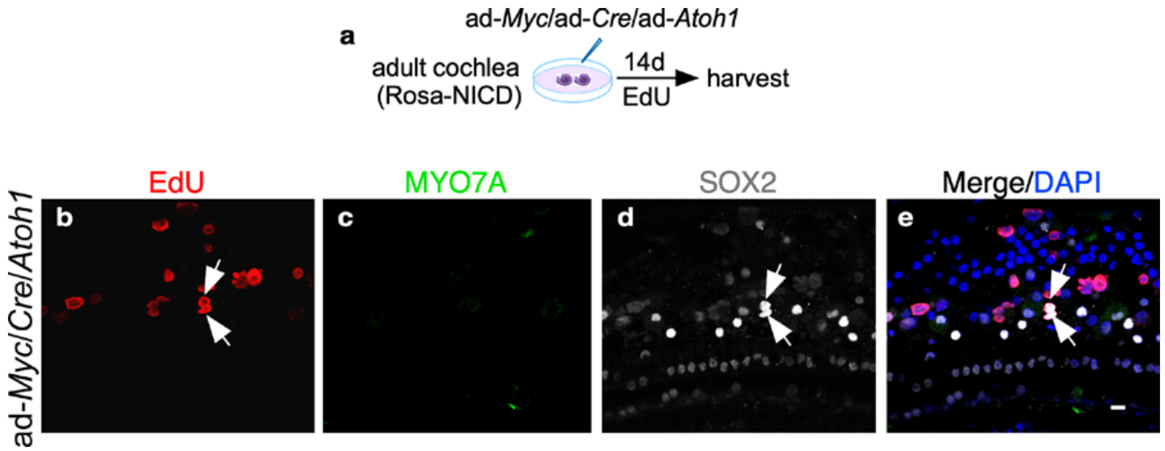

Supplementary Figure 6. **Continuous MYC/NICD activities failed to induce adult SC-to-HC transdifferentiation in response to *Atoh1* induction.** (**a**) A diagram illustrating the procedure. (**b-e**) 14 days after co-infection with ad-*Myc/*ad-*Cre/*ad-*Atoh1*, SCs labeled with EdU were detected (SOX2^{+}/ EdU^{+}, arrows) in cultured adult Rosa-NICD cochleae. No HCs (MYO7A^{+}) were detected. Scale bars: 10 μm.

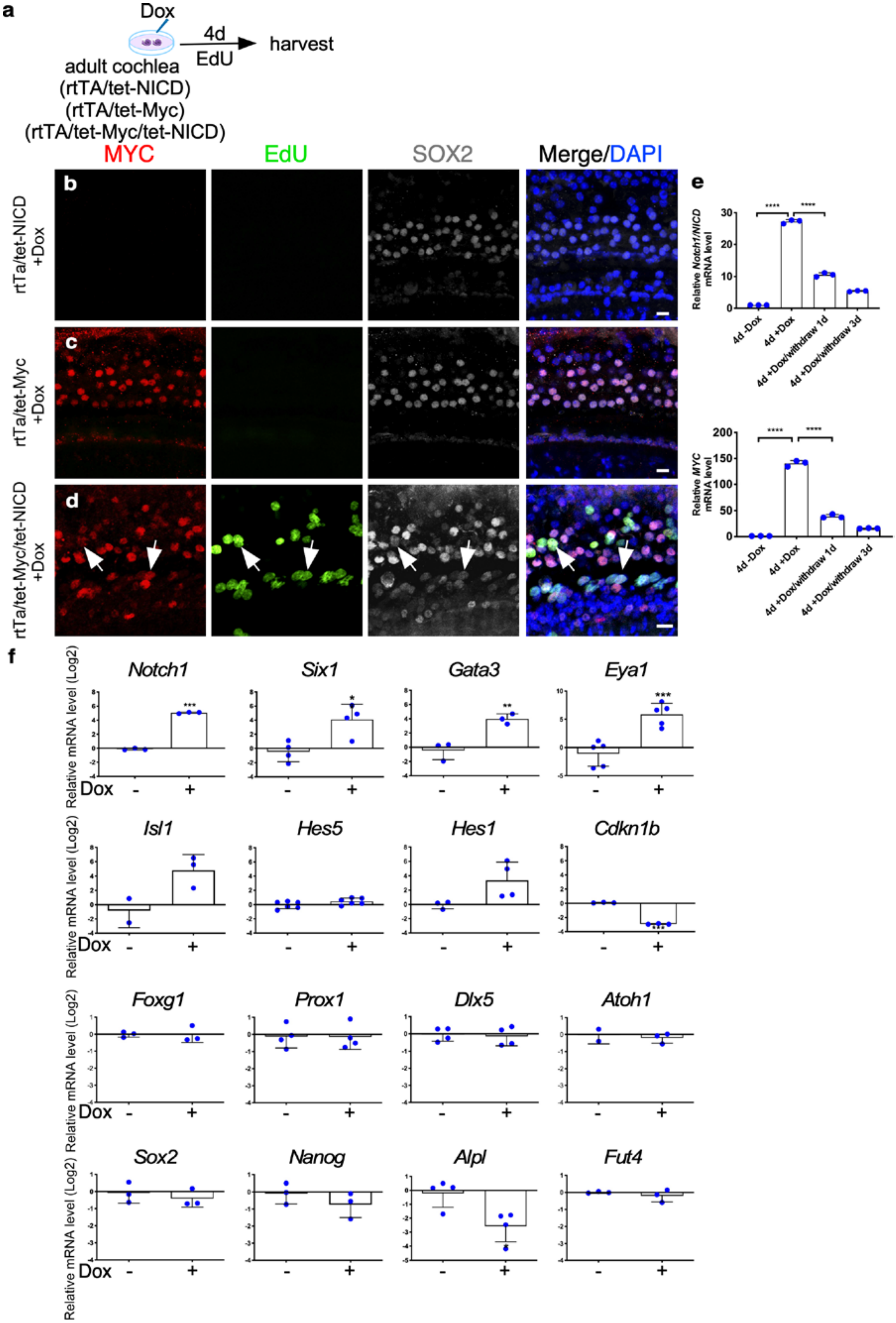
a
Dox
4d
EdU
harvest
adult cochlea
(rtTA/tet-NICD)
(rtTA/tet-Myc)
(rtTA/tet-Myc/tet-NICD)
MYC
EdU
SOX2
Merge/DAPI
b
c
d
rtTa/tet-NICD +Dox
rtTa/tet-Myc +Dox
rtTa/tet-Myc/tet-NICD +Dox
e
Relative Notch1/NICD mRNA level
Relative MYC mRNA level
4d -Dox
4d +Dox
4d +Dox/withdraw 1d
4d +Dox/withdraw 3d
f
Relative mRNA level (Log2)
Notch1
Six1
Gata3
Eya1
Isl1
Hes5
Hes1
Cdkn1b
Foxg1
Prox1
Dlx5
Atoh1
Sox2
Nanog
Alpl
Fut4
Dox

Supplementary Figure 7. **Transient and reversible *Myc/NICD* activation and re-activation of inner ear progenitor genes in adult cochlea.** (**a**) A diagram illustrating the study of different Dox-inducible mouse models for transient activation of *NICD*, *Myc* and *Myc/NICD*, respectively. (**b**) In cultured adult rtTA/tet-*NICD* cochlea following 3-day Dox treatment, no EdU^+ cell was detected in the sensory epithelial region. SOX2 labeled the SCs. (**c**) *Myc* was activated in the cultured adult rtTA/tet-*Myc* cochlea following 3-day Dox treatment, no EdU^+ cell was detected in the sensory epithelial region. (**d**) In cultured adult rtTA/tet-*Myc/tet-NICD* cochlea treated with Dox for 3 days, MYC^+/EdU^+ cells were detected in the sensory epithelial region, including dividing SCs (MYC^+/$SOX2^+$/EdU^+, arrows). (**e)** Reversible transactivation of c-*Myc* and *NICD* by qRT-PCR. Drastic up-regulation of *NICD* and *Myc* expression was detected four days after Dox exposure, and their rapid down-regulation three days following Dox withdrawal. ****$p<0.0001$, one-way ANOVA of Tukey's multiple comparison test. Error bar, mean ± s.d.; n=3 for each condition. (**f**) qRT-PCR, shown as expression level change at the log2 scale, in cultured adult rtTA/tet-*Myc*/tet-*NICD* mouse cochlea after 7-day Dox exposure. Up-regulation of inner ear progenitor genes included *Six1* (30-fold), *Eya1* (100-fold), *Gata3* (18-fold) and *Isl1* (48-fold), as well as *Notch1* (30-fold) and its target *Hes1* (26-fold). Some inner ear progenitor genes (*Hes5*, *Foxg1* and *Dlx5*) showed little expression level change. Stem cell genes (*Fut4*, *Sox2*, *Nanog* and *Alpl*) or differentiation genes (*Prox1*, *Cdkn1b* and *Atoh1)* were not up-regulated. *$p<0.05$; **$p<0.01$; ***$p<0.001$, two-tailed unpaired Student's t-test. Error bar, mean ± s.d., n=4. n is the number of biologically independent samples. Scale bars: 10 μm.

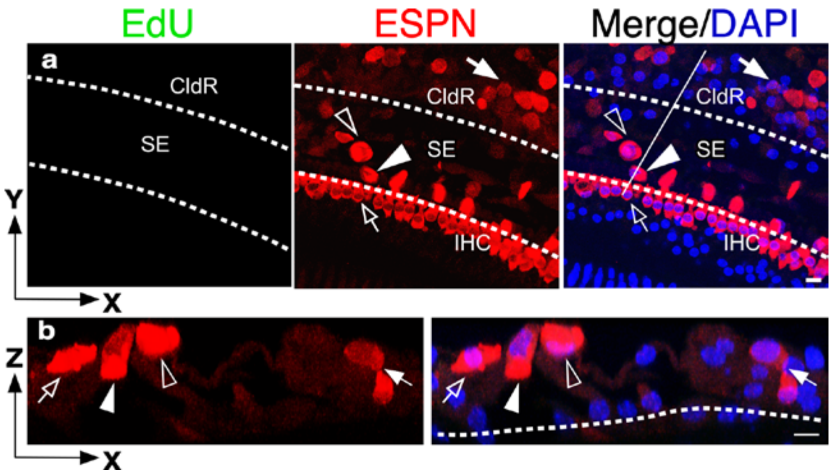

Supplementary Figure 8. *Atoh1*-induced HC regeneration in adult cochlea *in vivo* through transient activation of *Myc*/*NICD*. (**a**) Using the procedure as in Fig. 4a, 22 days after ad-*Atoh1* injection, ectopic HC-like cells (ESPN^{+}) were detected in the sensory epithelial region lateral to IHC (SE, between two dashed lines) and in the Claudius' cell region (CldR). The ectopic HC-like cells in the SE were likely from transdifferentiation of inner pillar cells (solid arrowhead) and outer pillar cells (open arrowhead), with the ESPN level comparable to the existing IHCs (open arrow). ESPN expression was weaker in some ectopic HC-like cells in the CldR region (arrow). (**b**) A cross section along the solid line in a to show relative positions of regenerated ectopic HC-like cells in relation to the existing IHC (open arrow). The markers indicated the same cells as in a. The dashed line demarcated the boundary between the SE cells and the basilar membrane. Scale bars: 10 μm.

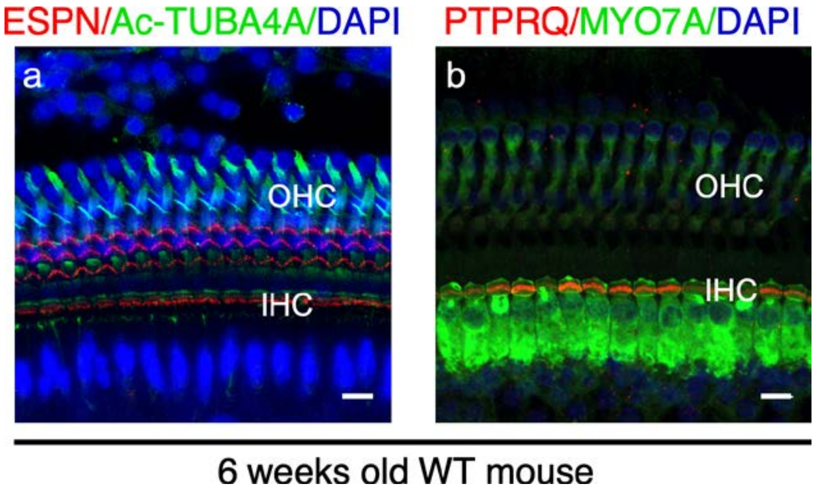

Supplementary Figure 9. **Markers for hair bundles in adult mouse cochlea.** (**a**) Acetylated-tubulin (Ac-TUBA4A) was not detected in the stereocilia in adult mouse cochlear HCs. (**b**) PTPRQ was detected only in stereocilia of IHCs but not OHCs in adult mouse cochlea. HCs were labeled with ESPN or MYO7A. Scale bars: 10 μm.

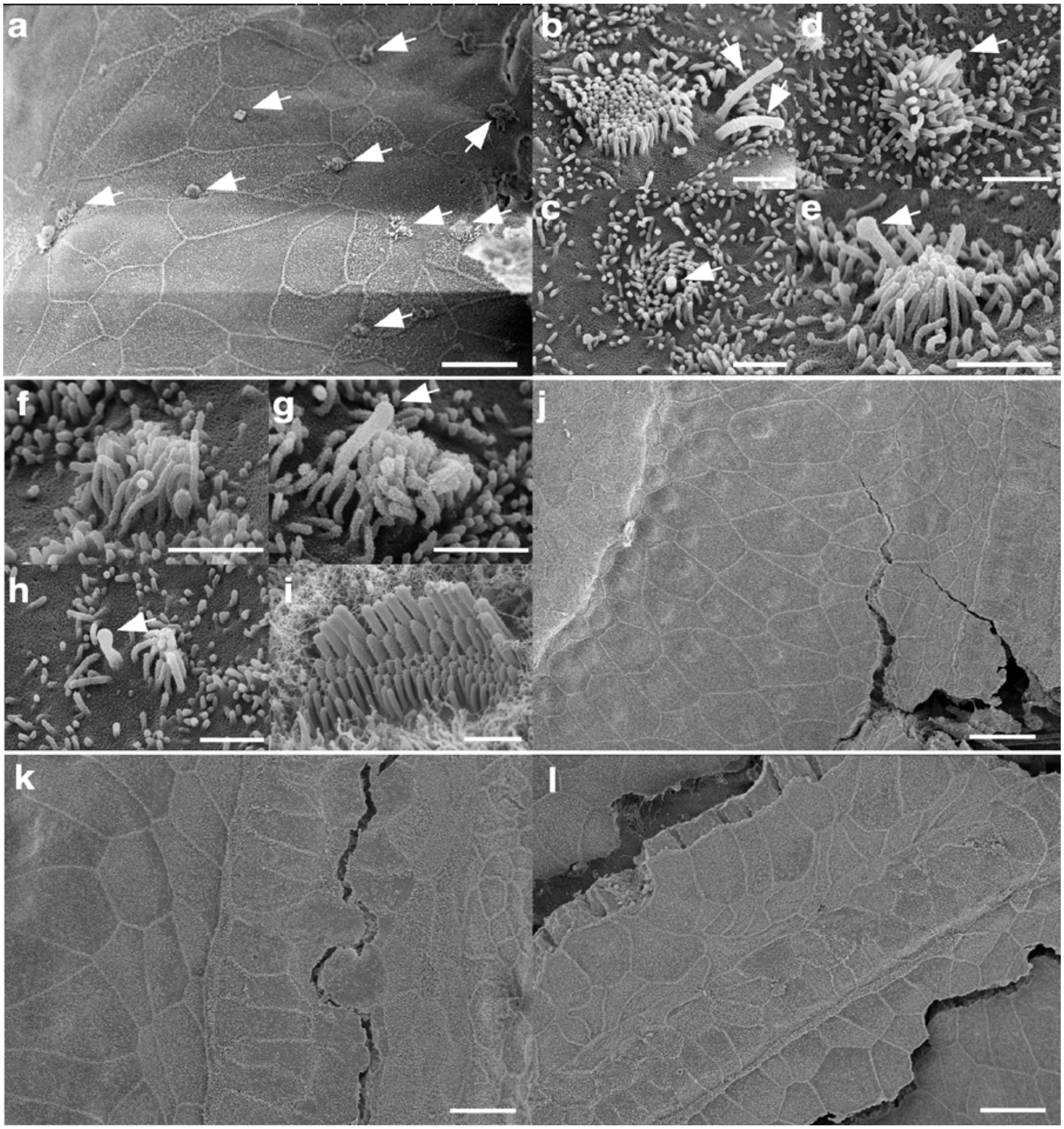

Supplementary Figure 10. **SEM images of regenerated HC-like cells in adult rtTA/tet-*Myc*/tet-*NICD* mouse cochlea *in vitro*.** (**a**) An overview of numerous ectopic HC-like cells in the limbus region of 6-week rtTA/tet-*Myc*/tet-*NICD* mouse cochlea 3 days after Dox treatment followed by ad-*Atoh1* infection for 14 days. (**b-h**) Images of

kinocilia (arrows) and immature stereocilia of regenerated HC-like cells. (**i**) Immature stereocilia in wild type P5 cochlea IHC. (**j**) In control untreated adult rtTA/tet-*Myc*/tet-*NICD* mouse cochlea in culture for 17 days, no HC-like cells with stereocilia were detected. (**k**) In control adult rtTA/tet-*Myc*/tet-*NICD* mouse cochlea treated with Dox for 3 days followed by 14 days in culture, no HC-like cells with stereocilia were detected. (**l**) In control adult rtTA/tet-*Myc*/tet-*NICD* mouse cochlea treated with ad-*Atoh1* for 14 days in culture, no HC-like cells with stereocilia were detected. Scale bars: 10 μm in a, f-h; 1 μm in b-e.

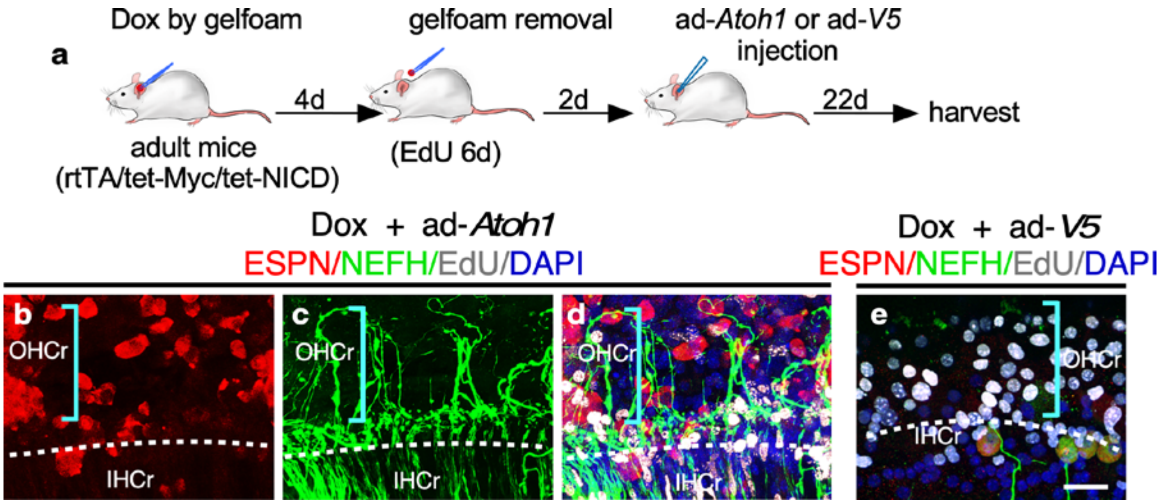

Supplementary Figure 11. **Regenerated HC-like cells recruited neural fibers from spiral ganglion neurons to form connections *in vivo*.** (**a**) A schematic diagram illustrating *in vivo* Dox induction of *Myc/NICD* and HC induction by ad-*Atoh1*. (**b-d**) The ganglion neurites (labeled with NEFH) were in contact with regenerated HC-like cells ($ESPN^+$) in the OHC region (OHCr). (**e**) In control of Dox-treated adult rtTA/tet-*Myc*/tet-*NICD* cochlea without ad-*Atoh1*induction, most neurites retracted with few remaining fibers in contact with the surviving IHCs. The dotted lines demarcated the boundary between the IHC and OHC regions. Scale bars: 20 μm.

SUPPLEMENTARY METHODS

Immunohistochemistry

Cochleae were fixed in 4% paraformaldehyde at 4°C overnight, followed by decalcification in 120 mM EDTA for 2-7 days. The decalcified cochlea was used for whole mount immunohistochemistry following a standard procedure [1]. The antibodies used are shown in Supplementary Table 1.

Supplementary Table 1. Antibodies used in this study

Antibodies	Species	Dilution	Source	Cat#
Acetylated tubulin (Ac-TUBA4A)	Mouse	1:500	Sigma-Aldrich	T6793
Alpha tubulin (TUBA4A)	Rabbit	1:200	Abcam	ab4074
AURKB	Mouse	1:200	BD Transduction Laboratories	611082
BrdU	Rat	1:200	ABD Serotech	OBT0030G
Cleaved Caspase 3 (CASP3)	Rabbit	1:200	BD Biosciences	559565
ESPN	Rabbit	1:200	Gift from J. Bartles	Northwestern Univ
JAG1	Rabbit	1:200	Santa Cruz Biotech	sc8303
MKI67	Rabbit	1:200	Thermo Scientific	RM9106S0
MYC (N-262)	Rabbit	1:200	Santa Cruz Biotech	sc-764
MYO7A	Rabbit	1:500	Proteus Biosciences	25-6790
NEFH	Chicken	1:1000	Millipore	AB5539
PVALB	Mouse	1:500	Sigma-Aldrich	P3088
pH3	Rabbit	1:100	Cell Signaling Technology	3377S
SLC26A5 (Prestin)	Goat	1:500	Santa Cruz Biotech	sc-22694
PTPRQ	Rabbit	1:200	Gift from Dr. Bowen-Pope	Univ of Washington
Phospho-rpS6	Rabbit	1:100	Cell Signaling Technology	2211S
S100A1	Rabbit	1:200	Sigma-Aldrich	HPA006462
SOX2	Goat	1:200	Santa Cruz Biotech	sc-17320
V5	Mouse	1:200	Invitrogen	R960-25
SLC17A8 (VGLUT3)	Guinea Pig	1:5000	Millipore	AB5421

All Alexa Fluor secondary antibodies were from Invitrogen, with 1:1000 dilution.
For EdU labeling, Click-IT EdU Imaging Kits (C10337 for Alexa Fluor 488, C10339 for Alexa Fluor 594, C10340 for Alexa Fluor 647) were purchased from Invitrogen. The reactions were performed following the manufacturer's instructions.

FM1-43 uptake

To study the presence of functional channels in HC-like cells regenerated *in vivo,* 6-week-old Rosa-NICD cochleae were injected with ad-*Myc*/ad-*Cre* as described. EdU was injected by i.p. daily for 5 days. Thirty five days after viral injection, cochleae were dissected and incubated with FM1-43FX (5 μM)(Life Technologies) for 30 seconds before wash and fixation with 4% PFA. The cochleae were subsequently decalcified and processed for immunolabeling. FM1-43FX uptake in HC-like cells regenerated *in vitro* followed the same protocol on the induced Rosa-rtTA/tet-*Myc*/tet-*NICD* cochlea cultures described above.

Confocal microscopy

Confocal microscopy was performed using a Leica TCS SP5 with Leica Application Suite Advanced Fluorescence (LAS AF) software V2.6.0. Sequential scanning with different laser channels was used for image acquisitions. Confocal images were processed using ImageJ package (www:\\imagej.nih.gov\ij). For Z-stacks, equal numbers of images of adjacent optical sections, 0.25 μm in thickness, were used for processing with identical

parameters, including median filtering and adjustment of brightness and contrast, between experimental and control groups.

Statistical analysis

The Prism 6 statistical package (GraphPad Software, Inc) was used in data processing. To count the number of SCs *in vitro*, we used the mid-apical region of adult Rosa-NICD cochleae infected with ad-*Myc*/ad-*Cre* due to its generally well preserved structure after dissection. The number of $SOX2^+$ cells was counted as SC number. The average number of $SOX2^+$ SCs per 100µm was calculated as SC density for each cochlea and was used for statistical analysis. ad-GFP infected Rosa-NICD cochleae were used as control. To count HC-like cells upon transient MYC/NICD followed by activation of Atoh1 *in vitro*, multiple regions from the apex (75-100% of the length of cochlear duct from the hook) were included. Briefly, new HC-like cells generated from Hensen cell region, Deiters' cell region, pillar cell region, inner phalangeal and inner sulcus cell region were all included in the "SE" region; the cells generated from limbus region were included in the "Lib" region.

To count the number of HCs upon *Myc*/*Notch1* activation *in vivo*, the mid-basal regions (25-50% of the length of cochlear duct from the hook) of adult Rosa-NICD cochleae injected with ad-*Myc*/ad-*Cre* and Dox/ad-*Atoh1* treated rtTA/tet-MYC/tet-NICD cochleae were used, as they are near the injection sites where IHCs were infected after cochleostomy. $MYO7A^+$ HCs were counted and the HC density was calculated as the

average number of HCs per 100 μm for each cochlea. Controls were ad-GFP-injected cochleae. Multiple cochleae were used for each counting and in statistical analysis. Cell counting was done 12 days after viral injection, to allow IHCs to complete cell cycle.

Data were presented as mean ± s.d. Two-tailed Student's t-test was used to compare two groups ($p<0.05$ was considered significant). ANOVA analysis with Tukey's multiple comparison test was used to compare three or more groups ($p<0.05$ was considered significant).

qRT-PCR

The cochlear tissues including the organ of Corti, spiral limbus, and lateral wall were dissected out from the temporal bones of cultured adult cochleae. Total RNA was extracted from cochleae using RNeasy Mini Kits from Qiagen (Valencia, CA). cDNA was synthesized using Supercript II reverse transcriptase (Invitrogen) with random primers. Real-time quantitative PCR amplification reactions were carried out using QuantiTeck SYBR Green PCR kit (Qiagen, Valencia, CA) on an ABI StepOnePlus system (Applied Biosystem) with StepOne software V2.3. The primers used for qRT-PCR are shown in Supplementary Table 2. All reactions were carried out in duplicates with the expression of the gene normalized using *Gapdh* as the endogenous housekeeping control gene. The relative expression level was calculated by the $2^{-\Delta\Delta Ct}$ method.

Supplementary Table 2. Primers for qRT-PCR

Gene Name	Primer Name	Primer Sequence	Expected Size	ID
Nanog	Primer F409<br>Primer R520	GCCCTGATTCTTCTACCAGTCC<br>ACAGTCCGCATCTTCTGCTT	112bp	NM_028016.2
Fut4 (Ssea1)	Primer F1627<br>Primer R1764	GCCCTTCCTTACCTGTCACC<br>GTGGTCTGCTCTTCATTGTTCC	138bp	NM_010242.3
Alpl	Primer_F1146<br>Primer_R1245	CTGACTGACCCTTCGCTCTC<br>CAATCCTGCCTCCTTCCAC	100bp	NM_007431.3
Prox1	Primer_F2331<br>Primer_R2473	CTCGCAGCTCATCAAGTGG<br>GGGCTCGGTATAGCTCACAA	143bP	NM_008937.2
Atoh1	Primer_F614<br>Primer_757	CCAGCAAACAGGTGAATGG<br>TGGACAGCTTCTTGTCGTTG	144bp	NM_007500.4
Hes1	Primer_F301<br>Primer_R450	ACGACACCGGACAAACCA<br>ATGCCGGGAGCTATCTTTCT	150bp	NM_008235.2
Hes5	Primer_F102<br>Primer_R240	TGCTCAGTCCCAAGGAGAAA<br>AGCTTGGAGTTGGGCTGGT	139bp	NM_010419.4
Cdkn1b (p27Kip1)	Primer_F863<br>Primer_R987	CGGTGCCTTTAATTGGGTCT<br>AGCAGGTCGCTTCCTCATC	125bp	NM_009875.4
Gapdh	Primer_F894<br>Primer_R1027	TGCGACTTCAACAGCAACTC<br>ATGAGGTCCACCACCCTGT	134bp	NM_008084.2
Dlx5	Primer_1_F<br>Primer_1_R	CGACCCTATGGCGTGTAACT<br>CAGGAAGCCGAGTTCTCCA	147bp	NM_198854.2
Eya1	Primer_1_F1780	GCCAGGACCTGAGCACATAC	107bp	NM_010164.2

	Primer_1_R1886	CATCCAGTCCACACCACCTC		
Six1	Primer_1_F804<br>Primer_1_R910	AGAACCGGAGGCAAAGAGAC<br>CCCTTCCAGAGGAGAGAGTTG	107bp	NM_009189.3
Isl1	Primer_1_F<br>Primer_1_R	AGGACAAGAAACGCAGCATC<br>GGGTTAGCCTGTAAACCACCA	139bp	NM_021459.4
Sox2	Primer_1_F1047<br>Primer_1_R1159	GACCAGCTCGCAGACCTACA<br>CCTCGGACTTGACCACAGAG	113bp	NM_011443.3
Notch1	Primer_2_F<br>Primer_2_R	TGGAGGACCTCATCAACTCAC<br>CGTTCTTCAGGAGCACAACA	120bp	NM_008714.3
Gata3	Primer_F1319<br>Primer_R1452	CTACCGGGTTCGGATGTAAGT<br>GCAGGCATTGCAAAGGTAGT	134bp	NM_008091.3
Foxg1	Primer_F<br>Primer_R	GCCAGCAGCACTTTGAGTTACA<br>CGGTGGAGAAGGAGTGGTT	115bp	NM_008241.2
MYC	Primer_F<br>Primer_R	GGCTCCTGGCAAAAGGTCA<br>CTGCGTAGTTGTGCTGATGT	119bp	NM_002467.5
**Semi-qRT-PCR**				
Hes5	Hes5-145f<br>Hes5-524r	TGGAGAAGATGCGTCGGGACC<br>AGCAGCAGCCTTAGCGGAGG	380bp	NM_010419.4
Gapdh	GAPDH For<br>GAPDH Rev	GTGGCCAAGGTCATCCATGACAAC<br>CATGAGGTCCACCACCCTGTTGCT	507bp	NM_008084.3
Notch1/NICD	NICD-45f<br>NICD-794r	CAAAGTGTCAGAGGCCAGCAAGAA<br>GCATTGACGTCAGCATGTGAGTTG	750bp	NM_008714.3

SUPPLEMENTARY REFERENCES

1. Huang, M., Kantardzhieva, A., Scheffer, D., Liberman, M.C. & Chen, Z.Y. Hair cell overexpression of Islet1 reduces age-related and noise-induced hearing loss. *J Neurosci* **33**, 15086-15094 (2013).
