# Supplementary material for: Renewed proliferation in adult mouse cochlea and regeneration of hair cells
Source: Nat Commun. 2019 Dec 4;10:5530. doi: 10.1038/s41467-019-13157-7 (PMC6892913; doi:10.1038/s41467-019-13157-7)
Supplement: Supplementary file 3 — Reporting Summary [file 41467_2019_13157_MOESM3_ESM.pdf]

nature research

Corresponding author(s): Zheng-Yi Chen

Last updated by author(s): Aug 30, 2019

Reporting Summary

Nature Research wishes to improve the reproducibility of the work that we publish. This form provides structure for consistency and transparency in reporting. For further information on Nature Research policies, see Authors & Referees and the Editorial Policy Checklist.

Statistics

For all statistical analyses, confirm that the following items are present in the figure legend, table legend, main text, or Methods section.

n/a	Confirmed	
☐	☒	The exact sample size (n) for each experimental group/condition, given as a discrete number and unit of measurement
☐	☒	A statement on whether measurements were taken from distinct samples or whether the same sample was measured repeatedly
☐	☒	The statistical test(s) used AND whether they are one- or two-sided<br>*Only common tests should be described solely by name; describe more complex techniques in the Methods section.*
☐	☒	A description of all covariates tested
☐	☒	A description of any assumptions or corrections, such as tests of normality and adjustment for multiple comparisons
☐	☒	A full description of the statistical parameters including central tendency (e.g. means) or other basic estimates (e.g. regression coefficient) AND variation (e.g. standard deviation) or associated estimates of uncertainty (e.g. confidence intervals)
☐	☒	For null hypothesis testing, the test statistic (e.g. F, t, r) with confidence intervals, effect sizes, degrees of freedom and P value noted<br>*Give P values as exact values whenever suitable.*
☒	☐	For Bayesian analysis, information on the choice of priors and Markov chain Monte Carlo settings
☒	☐	For hierarchical and complex designs, identification of the appropriate level for tests and full reporting of outcomes
☒	☐	Estimates of effect sizes (e.g. Cohen's d, Pearson's r), indicating how they were calculated

Our web collection on statistics for biologists contains articles on many of the points above.

Software and code

Policy information about availability of computer code

Data collection	Confocal images were acquired using a Leica TCS SP5 with Leica Application Suite X (LAS X) software. qRT-PCR was performed on an ABI StepOnePlus system (Applied Biosystem) with StepOne software V2.3.
Data analysis	Prism 6 from GraphPad was used for statistic analysis. ImageJ (NIH Image) was used for IHC/OHC/SC counting.

For manuscripts utilizing custom algorithms or software that are central to the research but not yet described in published literature, software must be made available to editors/reviewers. We strongly encourage code deposition in a community repository (e.g. GitHub). See the Nature Research guidelines for submitting code & software for further information.

Data

Policy information about availability of data

All manuscripts must include a data availability statement. This statement should provide the following information, where applicable:
- Accession codes, unique identifiers, or web links for publicly available datasets
- A list of figures that have associated raw data
- A description of any restrictions on data availability

All data generated or analyzed during this study are included in this published article (and its supplementary information files).

Field-specific reporting

Please select the one below that is the best fit for your research. If you are not sure, read the appropriate sections before making your selection.

☒ Life sciences ☐ Behavioural & social sciences ☐ Ecological, evolutionary & environmental sciences

Life sciences study design

All studies must disclose on these points even when the disclosure is negative.

Sample size	A minimum of 3 animals were used for statistical analyses in each experiment.
Data exclusions	Immunostaining data were excluded if the signal could not be distinguished from background noise, whereas the antibodies were known to work well. In this case we considered the experiment did not work.
Replication	All the experiments presented were replicated with the sample size and variation indicated in the figure legends and methods sections.
Randomization	The animals were randomly chosen for control and experimental groups.
Blinding	Counting of HCs and SCs were made without prior knowledge of genotype. Also a majority of counting was done by more than one people without prior knowledge of the experimental design.

Reporting for specific materials, systems and methods

We require information from authors about some types of materials, experimental systems and methods used in many studies. Here, indicate whether each material, system or method listed is relevant to your study. If you are not sure if a list item applies to your research, read the appropriate section before selecting a response.

Materials & experimental systems

n/a	Involved in the study	
☐	☒	Antibodies
☒	☐	Eukaryotic cell lines
☒	☐	Palaeontology
☐	☒	Animals and other organisms
☒	☐	Human research participants
☒	☐	Clinical data

Methods

n/a	Involved in the study	
☒	☐	ChIP-seq
☒	☐	Flow cytometry
☒	☐	MRI-based neuroimaging

Antibodies

Antibodies used

Primary Antibodies:
Acetylated tubulin, Mouse, 1:500, Sigma-Aldrich T6793; alpha tubulin, Rabbit, 1:200, Abcam ab4074; AurB, Mouse, 1:200, BD Transduction Laboratories 611082; BrdU, Rat, 1:200, ABD serotech OBT0030G; Esp, Rabbit, 1:200, Gift from J. Bartles, Northwestern Univ; JAG1, Rabbit, 1:200, Santa Cruz Biotech sc8303; Ki-67, Rabbit, 1:200, Thermo Scientific RM9106S0; MYC (N-262), Rabbit, 1:200, Santa Cruz Biotech sc-764; MYO7A, Rabbit, 1:500, proteus biosciences 25-6790; NF-H, Chicken, 1:1000, Millipore AB5539; PVALB, Mouse, 1:500, Sigma-aldrich P3088; Ph3, Rabbit, 1:100, Cell Signaling Technology 3377S; Prestin (SLC26A5), Goat, 1:500, Santa Cruz Biotech sc-22694; PTPRQ, Rabbit, 1:200, Gift from Dr. Bowen-Pope, Univ of Washington; Phospho-rpS6, Rabbit, 1:100, Cell Signaling Technology, 2211S; S100A1, Rabbit, 1:200, Sigma-Aldrich HPA006462; SOX2, Goat, 1:200, Santa Cruz Biotech sc-17320; V5, Mouse, 1:200, Invitrogen R960-25; VGLUT3, Guinea Pig, 1:5000, Millipore AB5421

Secondary Antibodies (all from Invitrogen):
Donkey anti-rabbit Alexa Fluor 488 (A21206), Alexa Fluor 594 (A21207) or Alexa Fluor 647 (A31573), donkey anti goat Alexa Fluor 594 (A11058) or Alexa Fluor 647 (A21447), donkey anti mouse Alexa Fluor 488 (A21202), Alexa Fluor 594 (A21203) or Alexa Fluor 647 (A31571), donkey anti-rat Alexa Fluor 594 (A21209), goat anti-chicken Alexa Fluor 488 (A-11039), goat anti-guinea pig Alexa Fluor 488 (A-11073).

Validation

All of the antibodies used in this study work well for immunofluorescence in adult mouse cochlea. Validation details of commercial antibodies are available from the manufacturer's websites. The staining of antibodies in the study is consistent with what is reported in previously published studies on the adult mouse cochlea and other tissues (Shu et al.,Hum Gene Ther. 2016;27(9):687-99; Huang et al., J Neurosci. 2013;33(38):15086-94; Sekerkova et al., J Comp Neurol. 2008;509:661-76; Goodyear et al., J Neurosci. 2003;23(27):9208-19)

Animals and other organisms

Policy information about studies involving animals; ARRIVE guidelines recommended for reporting animal research

Laboratory animals

All of the antibodies used in this study work well for immunofluorescence in adult mouse cochlea. Validation details of commercial antibodies are available from the manufacturer's websites. The staining of antibodies in the study is consistent with what is reported in previously published studies on the adult mouse cochlea and other tissues (Shu et al.,Hum Gene Ther. 2016;27(9):687-99; Huang et al., J Neurosci. 2013;33(38):15086-94; Sekerkova et al., J Comp Neurol. 2008;509:661-76; Goodyear

	et al., J Neurosci. 2003;23(27):9208-19)
Wild animals	The study did not involve wild animals.
Field-collected samples	The study did not involve samples collected from field.
Ethics oversight	The use of animals for the study and the protocols were approved by the Massachusetts Eye & Ear Infirmary IACUC committee. The use of virus was approved by the Harvard Medical School COMS Committee.

Note that full information on the approval of the study protocol must also be provided in the manuscript.
