# Supplementary material for: Renewed proliferation in adult mouse cochlea and regeneration of hair cells
Source: Nat Commun. 2019 Dec 4;10:5530. doi: 10.1038/s41467-019-13157-7 (PMC6892913; doi:10.1038/s41467-019-13157-7)
Supplement: Supplementary file 4 — Description of Additional Supplementary Files [file 41467_2019_13157_MOESM4_ESM.pdf]

Description of Additional Supplementary Files

File name: Supplementary Movie 1

Description: Robust regeneration of HC-like cells in the IHC and limbus regions in adult cochlea *in vivo*. A movie of 3D reconstruction of confocal z-stacked images from Fig. 4d to show supernumerary HC-like cells ($ESPN^{+}$, green) regenerated in the IHC and limbus regions (IHC, Lib). The bundles are visible as protrusions from some HC-like cells in the limbus region. Proliferation was shown by EdU (red) labeling.

File name: Supplementary Movie 2

Description: Regenerated HC-like cells likely form contact with the ganglion neurites. A movie of a 3D reconstruction of confocal z-stacked images of Supplementary Fig. 11d shows the ganglion neurites (NF, red) extending to the OHC region and wrapping around multiple regenerated HC-like cells (ESPN, green) transdifferentiated from SCs *in vivo*.
